# Supplementary figures and images for: Experimental-Evolution-Driven Identification of Arabidopsis Rhizosphere Competence Genes in Pseudomonas protegens
Source: mBio. 2021 Jun 8;12(3):e00927-21. doi: 10.1128/mBio.00927-21 (PMC8262913; doi:10.1128/mBio.00927-21)

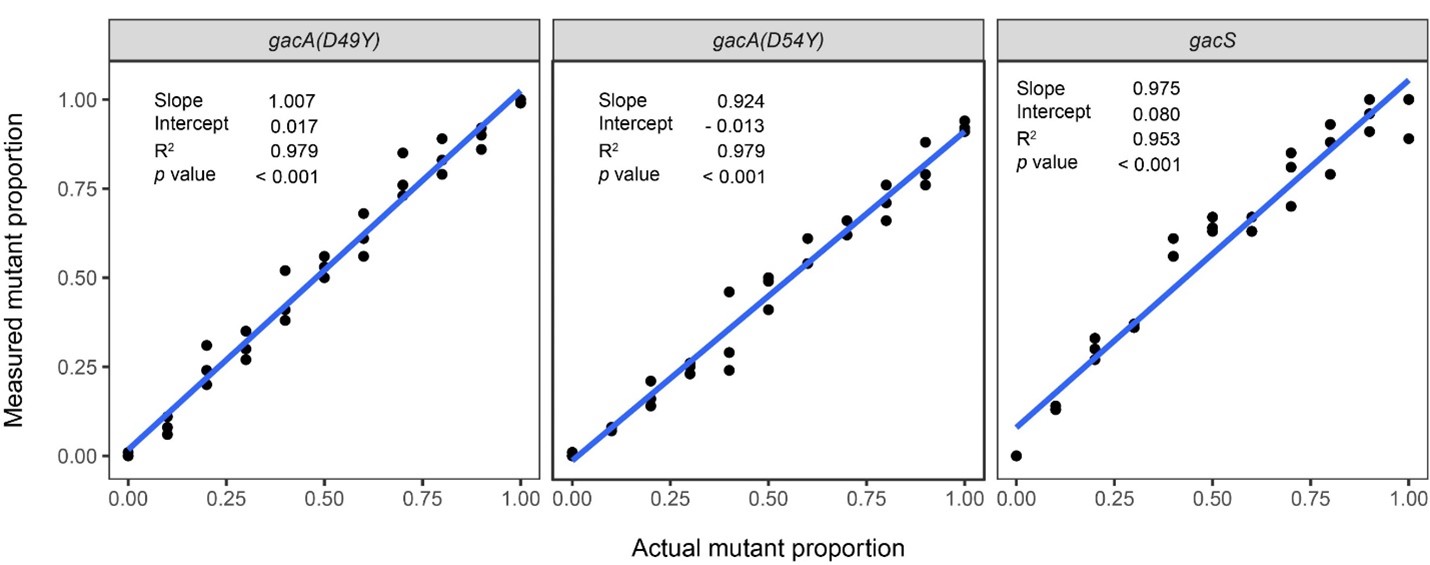

Supplement: FIG S1 [file mbio.00927-21-sf001.jpg]

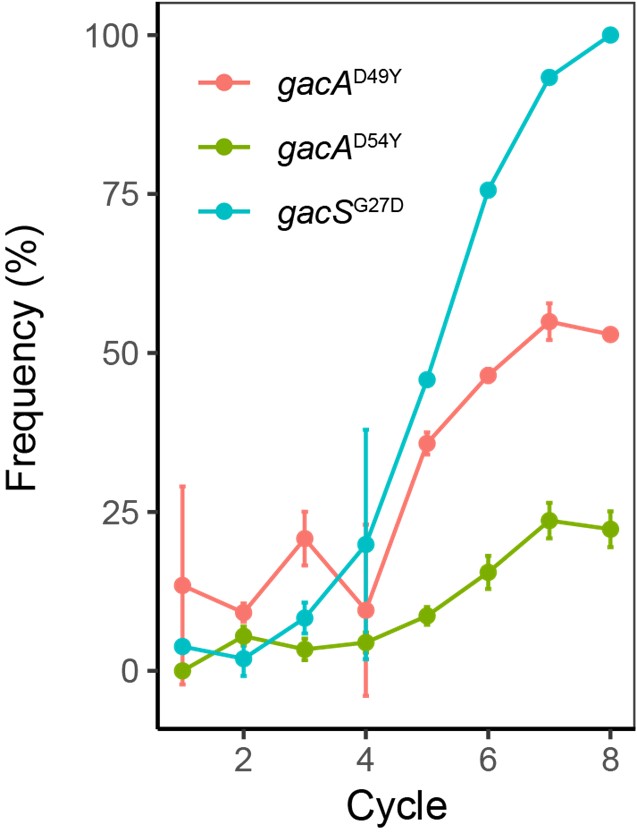

Supplement: FIG S2 [file mbio.00927-21-sf002.jpg]

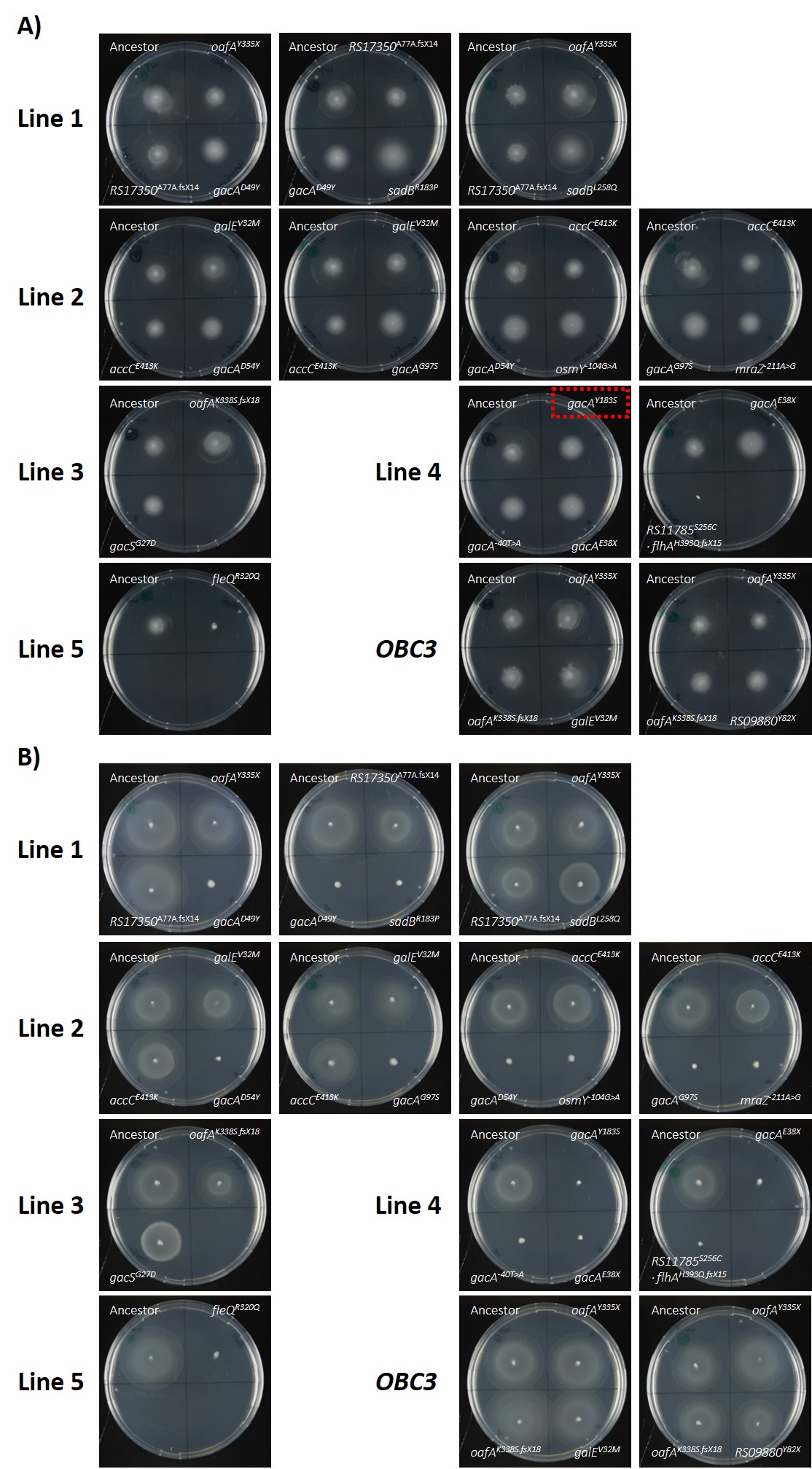

Supplement: FIG S3 [file mbio.00927-21-sf003.jpg]

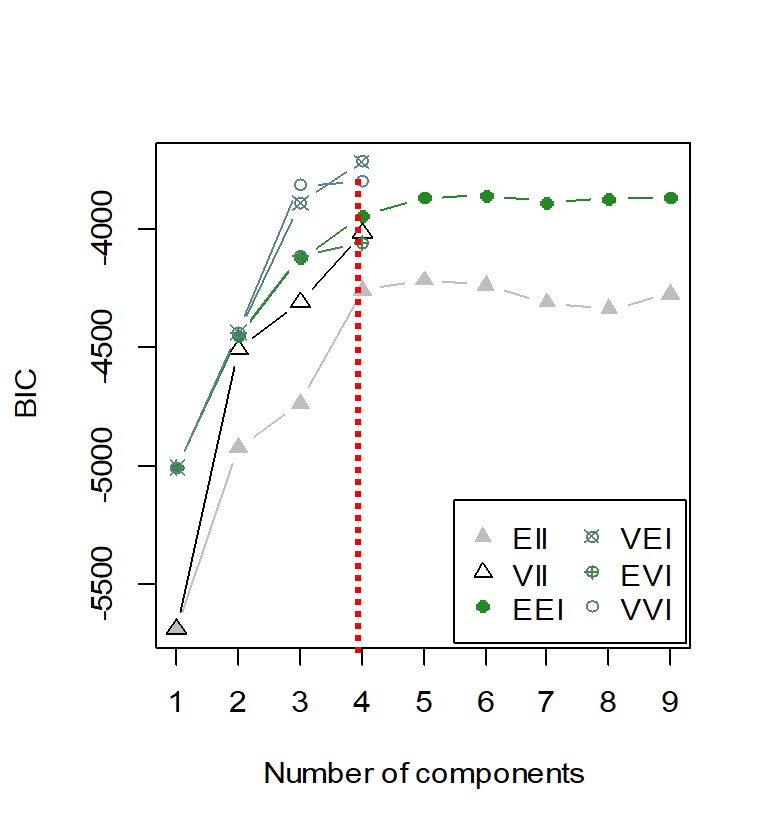

Supplement: FIG S4 [file mbio.00927-21-sf004.jpg]

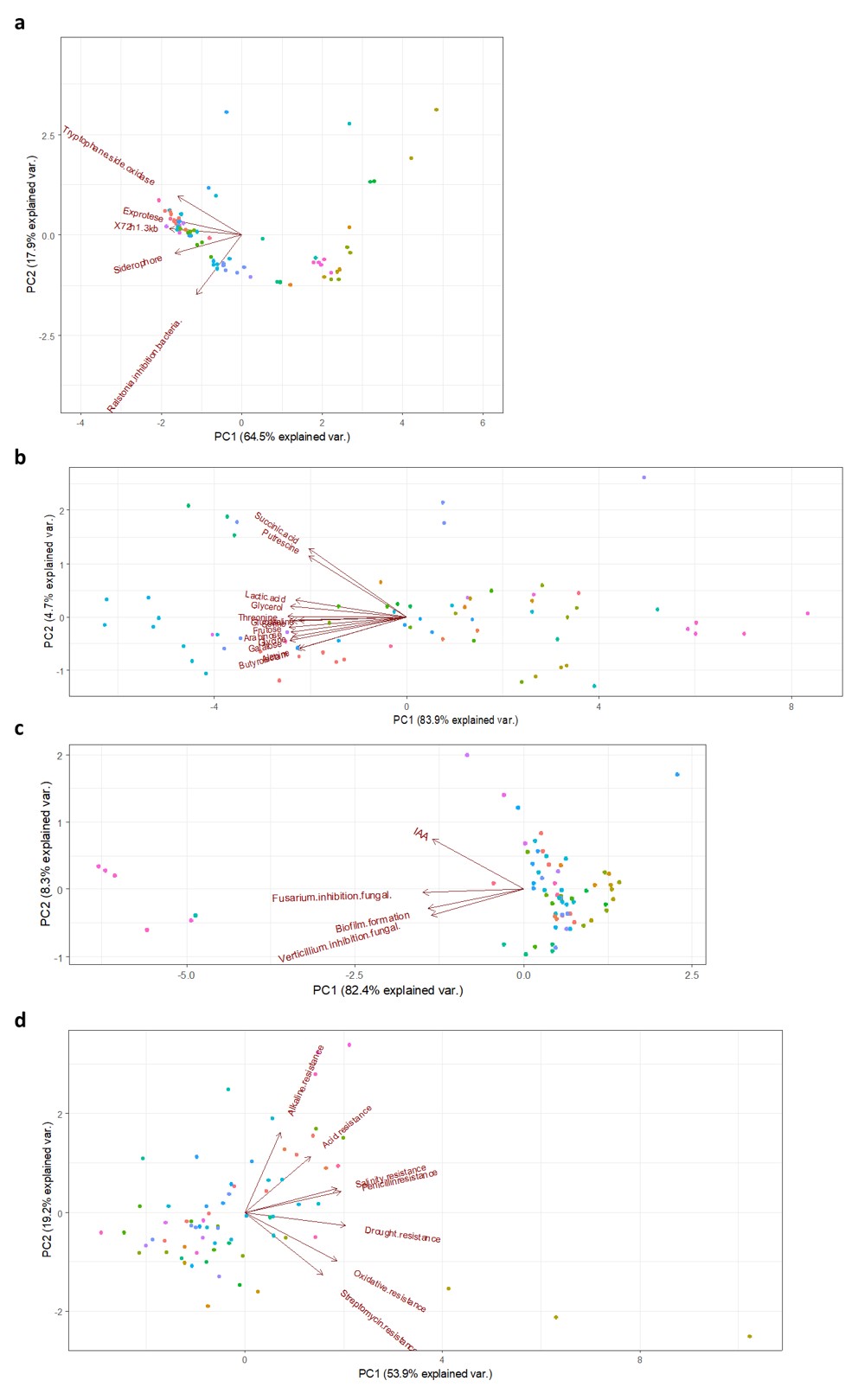

Supplement: FIG S5 [file mbio.00927-21-sf005.jpg]
